# Antiobesity Effect of a Novel Herbal Formulation *LI85008F* in High-Fat Diet-Induced Obese Mice

**DOI:** 10.1155/2021/6612996

**Published:** 2021-02-08

**Authors:** Hak Joo Choi, Hwa Young Kim, Kyoung Sik Park

**Affiliations:** ^1^Food Test and Research Center, Daejeon University, Daejeon 34520, Republic of Korea; ^2^Ju Yeong NS Co., Ltd, Seoul 05854, Republic of Korea; ^3^Department of Biomedical Science, Cheongju University, Cheongju 28503, Republic of Korea

## Abstract

A variety of natural products have been explored for their antiobesity potential and widely used to develop dietary supplements for the prevention of weight gain from excess body fat. In an attempt to find a natural antiobesity agent, this study was designed to evaluate the antiobesity activity of a novel herbal formulation *LI85008F* composed of extracts from three medicinal plants in high-fat diet- (HFD-) induced obese mice. After the thirteen-week oral administration of the test materials to mice, the body weight gain, whole-body fat mass, adipose tissue weight, and the expression levels of obesity-related proteins were measured. Our results indicated that *LI85008F* can suppress body weight gain and lower whole-body fat mass in HFD-induced obese mice. Significant decreases in epididymal and retroperitoneal fat mass were observed in *LI85008F*-treated groups compared with the HFD-fed control group (*p* < 0.05). Furthermore, the oral administration of *LI85008F* caused significant decreases in the expression level of adipogenic (C/EBP*α* and PPAR*γ*) and lipogenic (ACC) markers and notable increases in the production level of thermogenetic (AMPK*α*, PGC1*α* and UCP1) and lipolytic (HSL) proteins. These findings suggest that *LI85008F* holds great promise for a novel herbal formulation with antiobesity activities, preventing body fat accumulation and altering lipid metabolism.

## 1. Introduction

Obesity is a challenging health problem caused by the interaction of various genetic, dietary, lifestyle, and environmental factors [1]. The comorbidities associated with obesity include diabetes, hypertension, and cardiovascular diseases. Excess weight also has an enormous impact on the social, financial, and psychological status of obese individuals, which may contribute to the development of depression [2].

There are a variety of choices for obesity treatment, including life style changes, exercise, dietary control, weight-loss surgery, and prescription weight-loss medications [3]. Medications can facilitate weight loss in obese persons. The first class of medications developed for weight control causes symptoms that mimic the sympathetic nervous system, resulting in the major side effect of this class of medications as high blood pressure. These medications also lower appetite and increase a sensation of fullness. Another class of antiobesity medications suppresses appetite by increasing the concentration of neurotransmitters at the synaptic cleft, where hunger and fullness are regulated by brain neurotransmitters such as dopamine, serotonin, and norepinephrine [4]. Unfortunately, these weight-loss medications are known to have significant adverse effects including headache, insomnia, irritability, nervousness, abdominal pain, and diarrhea [5, 6]. In the face of adverse effects of synthetic drugs, the potential of natural products with antiobesity activity is under exploration [7]. Natural products including crude extracts and isolated pure natural compounds may serve as antiobesity agents via a variety of mechanisms including suppression of adipose tissue growth, inhibition of adipocyte differentiation, stimulation of lipolysis, and induction of apoptosis in existing adipocytes, thereby reducing adipose tissue mass [8].

*LI85008F* is composed of the extracts of three medicinal plants, *Moringa oleifera*, *Murraya koenigii*, and *Curcuma longa*. These medicinal plants were selected by screening hundreds of traditional herbal extracts for their ability to inhibit adipogenesis using a cell-based model [9]. *Moringa oleifera*, also known as drumstick tree, is indigenous to South Asia and India, and its leaves are highly nutritious and rich in amino acids, vitamins, minerals, and natural antioxidants [10]. The leaves of the plant have been traditionally used in the diet and for the treatment of various inflammatory-mediated diseases including cardiovascular diseases and diabetes [11]. *Murraya koenigii*, known as curry leaf, is used in folk medicine in India and other Asian countries as an analgesic, astringent, antidysenteric, febrifuge, hypolipidemic and hypoglycemic agent [12]. Turmeric (*Curcuma longa*) has a long tradition of being used as a spice by many cultures across the globe. This commonly consumed spice possesses antioxidant, anti‐inflammatory, anticancer, antigrowth, antiarthritic, antiaging, antiatherosclerotic, antidepressant, antidiabetic, antimicrobial, wound healing, and memory‐enhancing activities [13]. In traditional Indian medicine, turmeric has also been used to treat different ailments such as gynecological problems, gastric problems, hepatic disorders, infectious diseases, blood disorders, acne, psoriasis, dermatitis, rash, and other chronic ailments [14].

Although these spices have not traditionally been used for weight loss, the combination of *M. oleifera*, *M. koenigii*, and *C. longa* has been clinically shown to support body weight and fat loss in both obese and overweight individuals in a randomized, double-blind, placebo-controlled clinical trial [15]. In addition, Sengupta et al. [9] describe putative mechanisms for activity of this combination in modulating adipogenesis and lipolysis. The present study aimed to further investigate the antiobesity potential of *LI85008F* in a high-fat diet- (HFD-) induced obese mouse model.

## 2. Materials and Methods

### 2.1. Test Material

*LI85008F*, manufactured by Laila Nutraceuticals (Vijayawada, India) and provided by Ju Yeong NS Co., LTD. (Seoul, South Korea), is a novel herbal formulation composed of an aqueous ethanol extract of *Moringa oleifera* leaves, an aqueous alcohol extract of *Murrya koenigii* leaves, and *Curcuma longa* rhizome extract standardized to 95% total curcuminoids, mixed at a ratio of 6 : 3 : 1, respectively. Fresh plant raw materials were collected from local areas in Andhra Pradesh, India. Their voucher specimens are preserved in the Taxonomy Division at Laila Impex R & D Centre (Vijayawada, India). *LI85008F* is commercially available as Slendacor^®^ or Slimvance^®^.

### 2.2. Animals and Experimental Diets

Four-week-old male C57BL/6 mice (Raonbio, Yongin, South Korea) were maintained under a 12-hour light-dark cycle in a temperature- and humidity-controlled room during the experimental period. The mice were given laboratory pellet chow and water *ad libitum*. Following one week of acclimatization, 20 mice were randomly divided based on their body weight into four groups (*n* = 5) as follows: the normal group received a chow diet (ENVIGO, Huntingdon, UK); the control group was fed with a 60 kcal% fat high-fat diet (HFD; Research Diet Inc, Burnswick, USA); and the two experimental groups were provided with a HFD supplemented with 250 and 500 mg/kg of *LI85008F*, respectively. Mice allocated to the experimental groups were orally administered with *LI85008F* for 13 weeks. The body weight and feed consumption of each mouse were measured once a week and daily throughout the study duration, respectively. All animal care and use protocols following NIH guidelines were approved by the Institutional Animal Care and Use Committee (IACUC) of Daejeon University (approval No. DJUARB2020-003).

### 2.3. Determination of Body Fat Mass

The body fat mass of mice was measured using dual-energy X-ray absorptiometry (DXA) with an apparatus for small animals (Inalyzer, Medicors Inc., Seongnam, Korea). All scans were performed with the animals positioned prone and spread, with tape attached to each limb on the platform. The fat mass percentage was calculated as fat mass divided by total body weight and multiplied by 100.

### 2.4. Sampling Procedures

At the end of the 13-wk experimental phase, mice fasted overnight were anesthetized and sacrificed. The adipose tissues (epididymal and retroperitoneal fat pad) were harvested, weighed immediately, and stored at −80°C until further analysis.

### 2.5. Western Blot Analysis

Protein from each retroperitoneal fat sample was extracted with RIPA lysis and extraction buffer (Thermo Fisher Scientific, San Jose, USA) at 4°C. Then, the extracts were centrifuged at 13,200 r/min and 4°C for 30 min, and the supernatants of these tissues were used for western blotting analyses. The protein samples were separated on 10% SDS-PAGE gels and electrophoretically transferred onto PVDF membranes. The membranes were blocked at room temperature with 5% nonfat dry milk in TBST for 1 h and then incubated overnight at 4°C with the indicated primary antibodies (Cell Signaling Technology, Beverly, USA) as follows: AMP-activated protein kinase *α* (AMPK*α*; 1 : 100), acetyl-CoA carboxylase (ACC; 1 : 1,000), uncoupling protein 1 (UCP1; 1 : 1,000), CCAAT/enhancer binding protein *α* (C/EBP*α*; 1 : 1,000), hormone-sensitive lipase (HSL; 1 : 1,000), peroxisome proliferator-activated receptor-gamma coactivator 1*α* (PGC1*α*) (1 : 1,000), peroxisome proliferator-activated receptor *γ* (PPAR*γ*; 1 : 1,000), and *β*-actin (1 : 2,000). After washing three times with TBST, the blots were hybridized with secondary antibodies (1 : 10,000) conjugated to horseradish peroxidase. The proteins were visualized by enhanced chemiluminescence (Pierce Biotechnology, Rockford, USA) and analyzed using a Chemi-doc (Bio-Rad Laboratories, Hercules, USA).

### 2.6. Statistical Analysis

The results were expressed as mean ± standard deviation (SD). Statistical analyses were conducted using SPSS 21.0 (SPSS, Chicago, USA). The differences among groups were analyzed using one-way analysis of variance (ANOVA) with Duncan's test. Significance was set at *p* < 0.05 for all statistical analyses.

## 3. Results and Discussion

### 3.1. Effect of *LI85008F* on Body Weight Gain

**Figure 1:**
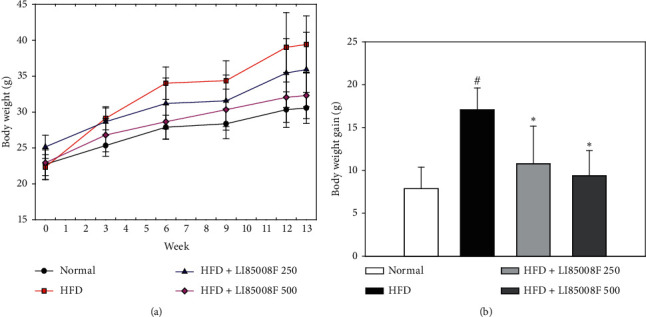
The effect of *LI85008F* on weekly body weight (a) and body weight gain (b) in mice for 13 weeks of treatment. Values are expressed as means ± SD (*n* = 5). The data were analyzed using ANOVA followed by Duncan's test. ^#^ and ^*∗*^ indicate the significant difference (*p* < 0.05) between normal vs. control group and control vs. *LI85008F* supplemented group, respectively.

The body weight and food intake of each mouse were measured on a weekly and daily basis throughout the study duration, respectively. Distinct separation can be seen from week 3 onwards, whereby the control group which received high-fat diet (HFD) revealed a remarkable increase in body weight when compared with the normal group fed with a regular diet, implying the induction of obesity in animals in the control group. From week 6 onwards, daily administration of test article *LI85008F* at the dose of each of 250 and 500 mg/kg significantly prevented the body weight gain in both *LI85008F*-treated groups (control: 17.08 ± 2.53 vs. *LI85008F* 250 : 10.78 ± 4.39, *p* < 0.05; *LI85008F* 500 : 9.37 ± 2.95, *p* < 0.05) (Figure 1).

### 3.2. Effect of *LI85008F* on Whole-Body Fat Mass

**Figure 2:**
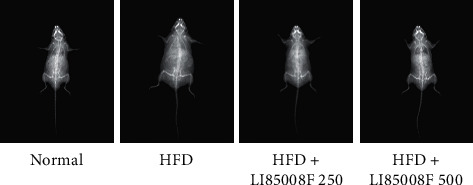
Representative images from DXA scans for mice in each group after 13 weeks of treatment.

To determine whether the observed decrease in body weight by treatment of *LI85008F* was due to a reduced accumulation of fat, we estimated whole-body fat mass by conducting dual-energy X-ray absorptiometry(DXA) with an apparatus for small animals (Figure 2).

**Figure 3:**
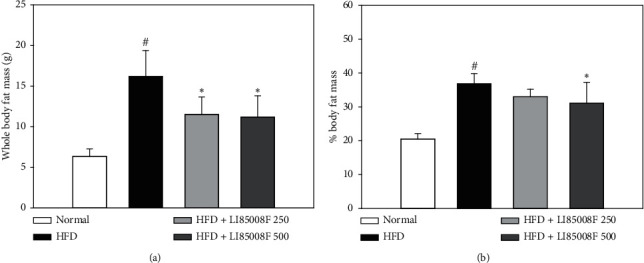
The effect of *LI85008F* on whole-body fat mass (a) and fat mass percentage (b) in mice for 13 weeks of treatment. Values are expressed as means ± SD (*n* = 5). The data were analyzed using ANOVA followed by Duncan's test. ^#^ and ^*∗*^ indicate the significant difference (*p* < 0.05) between normal vs. control group and control vs. *LI85008F* supplemented group, respectively.

The mice in the control group had a significant higher whole-body fat mass at the end of the 13-wk intervention period than in mice fed with a regular diet. In contrast, the daily oral administration of the test material *LI85008F* at the dose of each of 250 and 500 mg/kg resulted in a significantly reduced whole-body fat mass compared to that of the control group (control: 16.20 ± 3.18 vs. *LI85008F* 250 : 11.51 ± 2.16, *p* < 0.05; *LI85008F* 500 : 11.19 ± 2.63, *p* < 0.05) (Figure 3(a)). In addition, the fat mass percentage was calculated as fat mass divided by total body weight. In parallel with whole-body fat mass, significant decreases in fat mass percentage were found in mice treated with *LI85008F* at the dose of 500 mg/kg compared with the control group (control: 36.90 ± 2.95 vs. *LI85008F* 500 : 31.07 ± 6.20, *p* < 0.05) (Figure 3(b)).

### 3.3. Effect of *LI85008F* on Fat Mass of Adipose Tissue

**Figure 4:**
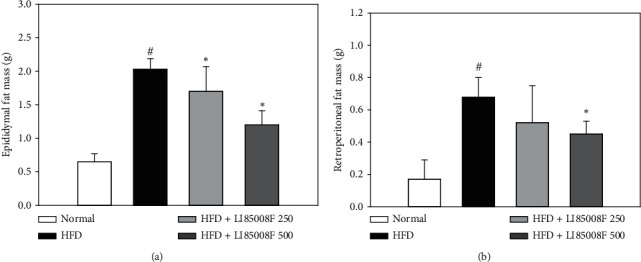
The effect of *LI85008F* on epididymal (a) and retroperitoneal fat pad weight (b) in mice for 13 weeks of treatment. Values are expressed as means ± SD (*n* = 5). The data were analyzed using ANOVA followed by Duncan's test. ^#^ and ^*∗*^ indicate the significant difference (*p* < 0.05) between normal vs. control group and control vs. *LI85008F* supplemented group, respectively.

After daily administration of test article *LI85008F* for 13 weeks, mice fed with HFD exhibited a significant increase in both the epididymal and retroperitoneal fat pad weights in comparison with the normal group. A significant decrease in epididymal fat pad weight was observed in all animals that received *LI85008F* at the dose of 250 mg/kg or 500 mg/kg for 13 weeks in a dose-dependent manner in comparison with the control group (control: 2.03 ± 0.16 vs. *LI85008F* 250 : 1.70 ± 0.37, *p* < 0.05; *LI85008F* 500 : 1.20 ± 0.21, *p* < 0.05) (Figure 4(a)). In retroperitoneal fat pad weight, there was a significant reduction in the group receiving 500 mg/kg of *LI85008F* compared to the control group (control: 0.68 ± 0.12 vs. *LI85008F* 500 : 0.45 ± 0.08, *p* < 0.05) (Figure 4(b)).

### 3.4. Effect of *LI85008F* on the Expression of Adipogenic and Lipogenic Markers in Adipose Tissue

**Figure 5:**
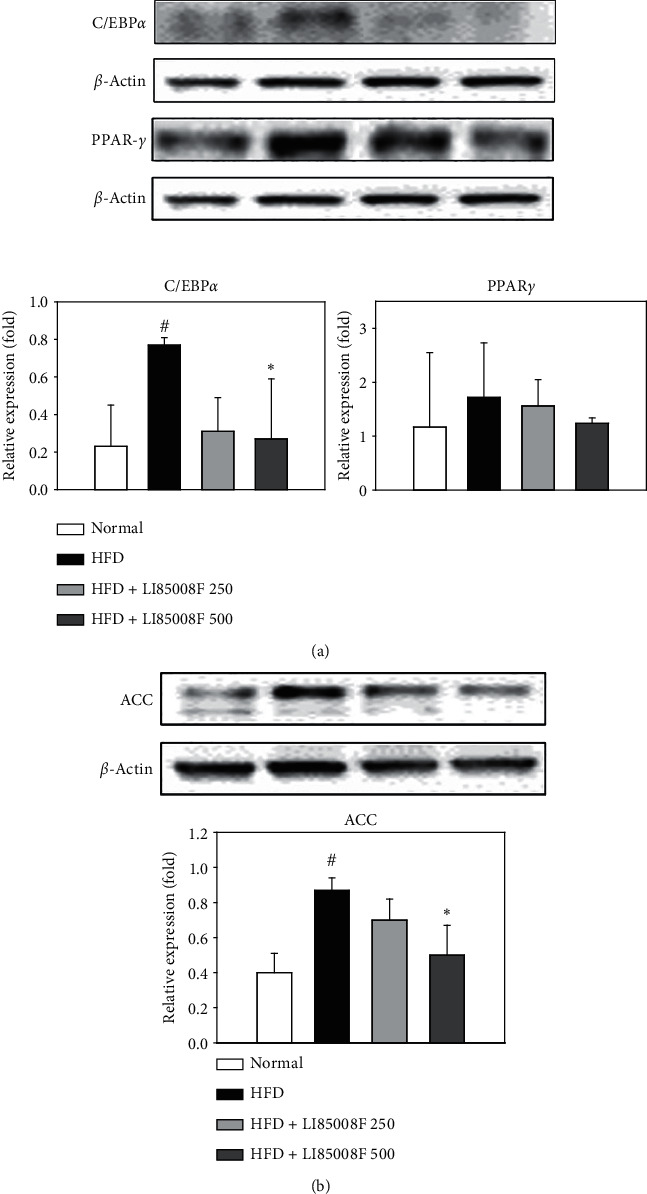
The effect of *LI85008F* on the expression level of adipogenic (a) and lipogenic markers (b) in retroperitoneal fat pads of mice for 13 weeks of treatment. Specific bands were quantified and are presented as bar graphs. Values are expressed as means ± SD (*n* = 5). The data were analyzed using ANOVA followed by Duncan's test. ^#^ and ^*∗*^ indicate the significant difference (*p* < 0.05) between normal vs. control group and control vs. *LI85008F* supplemented group, respectively.

To further elucidate the mechanism underlying the blockade of fat accumulation by *LI85008F* supplementation, the expression of the key markers of adipogenesis C/EBP*α* and PPAR*γ* in retroperitoneal fat pads was examined by western blot analysis. The adipogenesis process is tightly controlled by C/EBP*α*, which promotes the adipogenic pathway through activation of PPAR*γ* [16]. As shown in Figure 5(a), the expression of these two adipogenic markers was suppressed in *LI85008F*-treated mice compared to HFD-fed control mice. Moreover, the production level of a key lipogenic enzyme, ACC, which is regulated by C/EBP*α* or PPAR*γ* [17], was measured. The intake of *LI85008F* for 13 weeks significantly decreased the expression level of ACC in a dose-dependent manner (Figure 5(b)).

### 3.5. Effect of *LI85008F* on the Expression of Thermogenetic and Lipolytic Markers in Adipose Tissue

**Figure 6:**
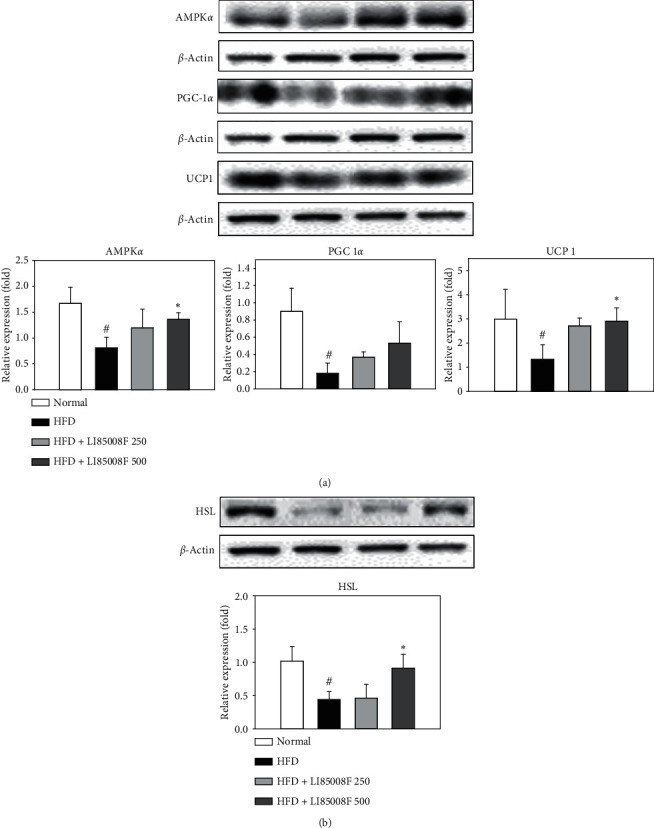
The effect of *LI85008F* on the expression level of thermogenetic (a) and lipolytic markers (b) in retroperitoneal fat pads of mice for 13 weeks of treatment. Specific bands were quantified and are presented as bar graphs. Values are expressed as means ± SD (*n* = 5). The data were analyzed using ANOVA followed by Duncan's test. ^#^ and ^*∗*^ indicate the significant difference (*p* < 0.05) between normal vs. control group and control vs. *LI85008F* supplemented group, respectively.

It has been reported that phosphorylation of AMPK*α* triggers the induction of gene expression of thermogenesis markers PGC1*α* and UCP1 [18]. Therefore, we evaluated the effect of *LI85008F* supplementation on the expression level of AMPK*α*, PGC1*α*, and UCP1 in retroperitoneal fat pads of HFD-fed mice. The oral administration of *LI85008F* for 13 weeks significantly up-regulated the production level of AMPK*α*, PGC1*α*, and UCP1 in a dose-dependent manner (Figure 6(a)). In addition, the alterations in the production level of lipase HSL involved in lipolysis by *LI85008F* intake were also investigated. *LI85008F*-treated mice showed higher protein expression of HSL than HFD-fed control mice (Figure 6(b), implying that *LI85008F* can be acting on the lipolytic pathway in HFD-induced obese mice.

Obesity can be defined as an increase in body weight beyond the limits of physical requirements, which results from an excessive accumulation of fat. Interestingly, in a previous clinical study [15], *LI85008F* supplementation for 8 weeks resulted in a significant body weight reduction in overweight or obese adults compared to those in the placebo group (*p* < 0.001), which is parallel with the suppressive effect of *LI85008F* on HFD-induced body weight gain in mice observed in this study (Figure 1). Furthermore, we have shown that *LI85008F* treatment significantly reduces whole-body fat mass and fat mass percentage using DXA (Figures 3(a) and 3(b)), most likely through the inhibition of fat accumulation in adipose tissue including epididymal and retroperitoneal fat pads (Figures 4(a) and 4(b)). The current study demonstrated that *LI85008F* suppressed adipogenesis and decreased lipid accumulation by inhibiting the expression of genes involved in adipogenesis and lipogenesis in adipose tissue (Figures 5(a) and 5(b)), which is consistent with the previous report [9] that *LI85008F* deactivates the key adipogenic transcription factors C/EBP*α* and PPAR*γ* in 3T3-L1 adipocytes.

As a master energy sensor, AMPK plays a key role in integrating hormones, nutrients, and stress signals to maintain whole-body energy homeostasis [19]. AMPK is activated by allosteric stimulation in response to an increased AMP/ATP ratio or mitochondria activity changes [20]. In skeletal and cardiac muscle, AMPK activation leads to the induction of gene expression of thermogenesis markers PGC1*α* and UCP1 [18]. In the present study, we demonstrated that *LI85008F* supplementation elevated AMPK*α* production level and induced the subsequent activation of PGC1*α* and UCP1 expression, leading to higher energy expenditure and lower body weight (Figure 6(a)). HSL, the major enzyme catabolizing triglycerides (TGs), induces lipolysis after phosphorylation of serine residues, leading to translocation of itself to the lipid droplets and markedly enhancing lipolysis [21]. As shown in Figure 6(b), *LI85008F* treatment to HFD-induced obese mice led to upregulate the expression of HSL involved in lipolysis pathways.

Based on these findings, we propose that *LI85008F* exerts antiobesity activities through a variety of mechanisms including suppression of adipogenesis, inhibition of lipogenesis, stimulation of lipolysis, and upregulation of thermogenesis and energy expenditure in adipose tissue, thereby reducing adipose tissue mass, whole-body fat mass, and body weight gain in HFD-induced obese mice.

## 4. Conclusions

The present study demonstrated that *L185008F* administration for 13 weeks significantly suppressed HFD-induced body weight gain and reduced whole-body fat mass, as well as fat weight of adipose tissue including epididymal and retroperitoneal fat in HFD-induced obese mice. These findings may be associated with significant decreases in the expression level of adipogenesis and lipogenesis proteins including C/EBP*α*, PPAR*γ*, and ACC, as well as marked upregulation in the production level of thermogenesis and lipolysis markers such as AMPK*α*, PGC1*α*, UCP1, and HSL induced by *L185008F* treatment in HFD-induced obese mice.

## Data Availability

All data generated or analyzed during this study are available and included in this article.
